# Time-Series Study of Associations between Rates of People Affected by Disasters and the El Niño Southern Oscillation (ENSO) Cycle

**DOI:** 10.3390/ijerph16173146

**Published:** 2019-08-28

**Authors:** Holly Ching Yu Lam, Andy Haines, Glenn McGregor, Emily Ying Yang Chan, Shakoor Hajat

**Affiliations:** 1 Jockey Club School of Public Health and Primary Care, The Chinese University of Hong Kong, Hong Kong, China; 2 Department of Public Health, Environments and Society, London School of Hygiene and Tropical Medicine, London WC1H 9SH, UK; 3 Centre for Climate Change and Planetary Health, London School of Hygiene and Tropical Medicine, London WC1H 9SH, UK; 4 Department of Geography, Durham University, Durham DH1 3LE, UK

**Keywords:** El Niño Southern Oscillation, natural disasters, number of people affected, El Niño, La Niña, Oceanic Niño Index (ONI)

## Abstract

The El Niño Southern Oscillation (ENSO) is a major driver of climatic variability that can have far reaching consequences for public health globally. We explored whether global, regional and country-level rates of people affected by natural disasters (PAD) are linked to ENSO. Annual numbers of PAD between 1964–2017 recorded on the EM-DAT disaster database were combined with UN population data to create PAD rates. Time-series regression was used to assess de-trended associations between PAD and 2 ENSO indices: Oceanic Niño Index (ONI) and multivariate El Niño Index (MEI). Over 95% of PAD were caused by floods, droughts or storms, with over 75% of people affected by these three disasters residing in Asia. Globally, drought-related PAD rate increased sharply in El Niño years (versus neutral years). Flood events were the disaster type most strongly associated with El Niño regionally: in South Asia, flood-related PAD increased by 40.5% (95% CI 19.3% to 65.6%) for each boundary point increase in ONI (*p* = 0.002). India was found to be the country with the largest increase in flood-related PAD rates following an El Niño event, with the Philippines experiencing the largest increase following La Niña. Multivariate ENSO Index (MEI)-analyses showed consistent results. These findings can be used to inform disaster preparedness strategies.

## 1. Introduction

The El Niño Southern Oscillation (ENSO) is a major driver of climatic variability that can have far reaching impacts across the globe. The ocean warm phase of the cycle, termed El Niño, means ‘little boy’ in Spanish and refers to the Christ child since its appearance was historically detected around December time. 

Both El Niño and the opposing ocean cool phase, La Niña (‘the little girl’), can significantly influence global climate patterns. El Niño and La Niña affects sea surface temperatures (SSTs) along the equatorial region of the Pacific Basin, which disrupts tropical atmospheric and ocean circulation patterns and modifies the mid-latitude jet stream [1]. This results in a range of environmental impacts in tropical and extra-tropical regions with altered temperature [2,3], precipitation patterns and extremes [4] which bear important public health consequences for many regions of the world [5,6]. The World Health Organization reported that the El Niño event of 2015/16 affected more than 60 million people worldwide [7], including contributing to the Zika outbreak in Latin America in 2016 [8] as well as dengue in Southeast Asia and Brazil [9], cholera in Tanzania [9], and hanta virus and plague in western U.S. [9]. There are also well established links between ENSO and other known climate-sensitive infectious diseases such as cholera and malaria [10,11]. The likelihood of natural disasters such as droughts and floods may also increase during El Niño or La Niña events [12,13,14]. A review summarising correlations between extreme atmospheric hazards and global teleconnections found ENSO to have significant connections with 15 regional hazards [15].

The public health consequences of natural disasters include undernutrition, injuries, mental health effects, and compromised healthcare supplies and hygiene conditions [16]. Regional variations in health impacts can reflect differences in the underlying vulnerabilities of populations due to differences in socio-economic and demographic profiles, as well as other factors such as geographical features [17]. Despite evidence showing teleconnections between ENSO and the occurrence of natural disasters [12,13,14,15], the resulting health impacts of such linkages are less well established. A simulation study reported that both the warm and cool phases of ENSO increased flood-related risks from an economic and urban damage perspective [18]. A study from 1995 indicated that the global impact of droughts (defined as events when foreign assistance was required) was more frequent during the two years period after warm ENSO events [13]. In another global assessment from 1997, there were indications that annual rates of people affected by natural disasters are linked to El Niño years [19]. The associations were strongest in the same year and the year following El Niño and the effect was most prominent in South Asia. However, it remains unclear whether disaster-related global health impacts are related to ENSO events in more recent decades against the backdrop of a rapidly changing climate. This knowledge gap calls for impact-based assessments to be conducted with updated data, however to the best of our knowledge this has not been undertaken to date.

To mark the latest International Day for Disaster Reduction held on 13 October, 2018, a report released by the United Nations Office for Disaster Risk Reduction (UNISDR) entitled Economic Losses, Poverty and Disasters: 1998–2017, headlined some sobering statistics; disasters killed 1.3 million people and affected 4.4 billion people between 1998–2017 [20]. Direct economic losses were valued at U.S.$ 2908 billion, 77% of which were due to climate-related disasters; and people exposed to natural hazards in the poorest nations were more than seven times more likely to die than equivalent populations in the richest nations [20]. This stark illustration of the need for better disaster preparedness, allied with the possibility that climate change may alter future ENSO patterns in unpredictable ways, including the likelihood of increasing the areas impacted [21], means that there is a pressing need to understand the links between ENSO and the numbers of people affected by natural disasters, and to identify the timing and locations where these impacts are likely to be greatest. By applying time-series regression techniques to extended datasets from the EM-DAT International Disaster Database, we characterised annual relationships between two ENSO indices, the Oceanic Niño Index (ONI) and the multivariate El Niño Index (MEI), and disaster burdens at the global, regional and country level between 1964–2017.

## 2. Materials and Methods

### 2.1. Disaster Databases

Annual numbers of people affected by natural disasters at the global, regional and country level between 1964–2017 were retrieved from EM-DAT. EM-DAT is an emergency events database launched and managed by the Centre for Research on the Epidemiology of Disasters (CRED), School of Public Health, Université catholique de Louvain, Brussels, Belgium [22]. Although the term ‘natural disaster’ implies that such disasters are unavoidable, we acknowledge that the occurrence and impact of such events may also depend on various social and other vulnerability factors; however we use the term here to distinguish them from technological disasters which are also recorded on EM-DAT. The main sources of the database include the United Nations (UN), and both governmental and non-governmental agencies. Disasters are recorded on the database if they fulfil at least one of the following criteria: (1) 10 or more people reported killed, (2) 100 or more people being affected, (3) declaration of a state of emergency, or (4) call for international assistance. People “affected” by a disaster is defined as “people requiring immediate assistance during a period of emergency”. To focus on health impacts, (including physical health, mental health and well-being according to the WHO [23]), we considered the “total affected” as the outcome measure in this analysis, which is the sum of the injured, homeless and “affected” categories.

The global analysis was conducted to provide an overview of the overall impacts of ENSO. Among the 15 disaster types listed in the EM-DAT category of ‘natural disasters’, floods, droughts and storms accounted for over 95% of the total people affected during the study period (Table 1), and these occurred mainly in East Asia, South Asia and Southeast Asia. We therefore focused our analysis on these three disaster types and three regions. Since the association between ENSO and natural disasters may vary within region [15], we further assessed associations at country-level in the three chosen regions. Rates of people affected by disasters (PAD) per 1000 people in the population were calculated using UN population data [24] to account for potential yearly fluctuations in global and regional population sizes [19]. The EM-DAT database only records yearly numbers of people affected by country and so it was not possible to assess associations with ENSO at a sub-annual or at a sub-national level.

For the regional analysis, droughts were ultimately not assessed due to less than 30 years during the study period recording events. The non-event years represent years with missing information rather than years with zero events. Countries in the three regions of interest with disasters occurring in at least 30 of the study years were included in the country-level analysis. Bangladesh, China, India, Indonesia, Pakistan, the Philippines, Sri Lanka, Thailand and Vietnam were included in the analysis of flood events. Bangladesh, China, Hong Kong, India, Japan, the Philippines, South Korea and Vietnam were assessed in relation to storms. Drought events were fewer and so not analysed at country-level.

### 2.2. Niño Years and ENSO Indices

For simple comparison of PAD rates between El Niño, La Niña and neutral years, El Niño and La Niña years were loosely defined in two ways to take account of the fact that ENSO events generally span calendar years. Annual PAD rates were firstly considered in years when the El Niño event began in the second half of the calendar year prior to the disaster year and ended within the first half of the same year as the disaster year. Secondly, annual PAD rates were considered in years when the El Niño event began in the second half of the same year as the disaster year and ended in the first half of the following year.

For the regression analyses, the two most commonly used ENSO indices [6], the Oceanic Niño Index (ONI) and the multivariate El Niño Index (MEI), were obtained from the National Oceanic and Atmospheric Administration (NOAA) [25]. ONI is a three-month moving average of sea surface temperature (SST) anomalies in the Niño 3.4 region in the equatorial Pacific which is 0.5 °C above or below the average for that time of the year [1]. Although countries may define El Niño events differently based on local climate conditions, SST anomalies ≥±0.5 °C in the Niño 3.4 region for several consecutive months is commonly used as an indicator of ENSO events [26,27,28]. In this study, we adopted the NOAA definition that an El Niño (or La Niña) event occurs when the ONI is higher (lower) than 0.5 °C (−0.5 °C) for at least five consecutive overlapping three-month periods during July-June [29]. The intensity of El Niño events was further categorized as very strong (ONI ≥2 °C), strong (1.5–1.9 °C), moderate (1.0–1.4 °C) and weak (0.5–0.9 °C), and similarly for La Niña events based on the corresponding negative values [29]. Years with ONI values between 0.5 °C and −0.5 °C are described as neutral years. MEI is a two-month seasonal index which integrates six variables in the tropical Pacific; these variables are sea-level pressure, zonal and meridional components of the surface wind, sea surface temperature, surface air temperature and total cloudiness fraction of the sky [25]. Similar to ONI, the index indicates oceanic and climatic anomalies; positive (or negative) values indicate an El Niño (La Niña) event, and the higher the value the stronger the ENSO phase intensity. The scale and range of MEI are also comparable to those of the ONI. Since El Niño events tend to become apparent at the tail-end of calendar years [30], the ENSO index during the period December to February for ONI (DJF) and December to January for MEI (DJ) were chosen to represent ENSO intensity and were assessed in relation to disasters only during the years subsequent to the December periods. Both indices (DJF and DJ) ≥+0.5 °C (≤−0.5 °C) were defined as the warm phase (cool phase) of ENSO. As the intensity of ONI in other months may also be an important consideration, we also considered a 12-month average of ONI (from July in the previous year to June in the same year as the disaster year) in sensitivity analysis.

The datasets analysed are available in the EM-DAT [22] and NOAA [25] repositories.

### 2.3. Statistical Analysis

For the descriptive analysis, the PAD rate was calculated by dividing the number of people affected by the population size in the calendar year (and expressed as a rate per 1000). To calculate the standardized yearly PAD rate, PAD series were first de-trended using cubic splines of time with two degrees of freedom (df). This was done to allow for gradual changes in PAD rates unrelated to annual ENSO activity, such as changes in disaster reporting rates, improvements in technology, infrastructure and resilience, and other secular changes. The choice of df = 2 aimed to remove the main trends without filtering out the short-term fluctuations that may be ENSO-related.

For the regression analysis, the combination of Poisson Generalized Additive Models (GAMs) and distributed-lag non-linear models for time-series data were used to flexibly model nonlinear and lag effects. These models allow for the identification of acute associations between exposure and outcome after control for long-term fluctuations. Effects of other potential confounders that display different time patterns are thus implicitly adjusted for. PAD was regressed over each ENSO index (ONI or MEI) one at a time with offset of the corresponding population size and the long-term time trend adjustment. Each ENSO index was modelled as a cross-basis term [31], a built-in function of the dlnm() in R. This function allows the outcome variable to be associated with both the time variable and the exposure variable simultaneously. A lag of up to 4 years was used to assess delayed effects. The df chosen (between two and five) were based on the model performance which is indicated by the General Cross Validation (GCV) score and the robustness of associations. The df chosen for the lag distribution and exposure were three and two in the global and country analysis respectively, whilst df = 4 for lag and df = 2 for exposure were used in the regional analysis. The long-term trend was adjusted for using a cubic spline term with df = 2. The maximum yearly lags used to assess cumulative relative risks were determined by examining effects of individual lags in the distributed-lag non-linear models. Cumulative relative risks were quantified by comparing risk against zero values of ONI or MEI. All statistical analyses were conducted using the mgcv() [32] and dlmn() [31] packages in R 3.3.1 (R Core Team, Vienna, Austria) [33] and IBM SPSS Statistics Version 22 (IBM Corp., New York, NY, USA) [34]. 

## 3. Results

### 3.1. Descriptive Statistics

Based on the EM-DAT database, 7.85 billion people were affected by natural disasters globally during the study period 1964–2017 (Table 1). The median yearly rate of people affected by natural disasters was 19.20 (per 1000 people). Over 95% of the people affected were impacted by floods, droughts or storms. More than 75% of people affected by the three disaster types occurred in Asia. South Asia contributed to over half of all drought-related PADs, whilst most flood- and storm-related PADs occurred in East Asian countries. Due to the high proportion of people affected by the disaster types in Asia, disaster-specific analyses were restricted to these disaster types, and regional and country-level analyses were conducted for East Asia, Southeast Asia and South Asia only. Descriptive statistics of disaster events at the country-level are presented in Table A1 online. China, India and Bangladesh had the highest numbers of people affected by floods during the study period. In terms of flood-related PAD rates (per 1000), Bangladesh was highest (mean PAD rate 76.92), followed by China (42.61) and Thailand (30.61). China and the Philippines had the highest number of storm related PAD. However, the Philippines reported storm-related PAD in 52 of the 54 study years and reported the highest PAD rate (per 1000) of 42.93. No individual countries reported drought in more than 30 years of the study period. The Oceanic Niño Index (ONI) (December-January-February (DJF)) and multivariate El Niño Index (MEI) (December-January (DJ)) measures were both normally distributed. The two indices were very highly correlated with each other: Pearson correlation coefficient of 0.94 (*p* < 0.005).

### 3.2. Global Analysis

**Figure 1:**
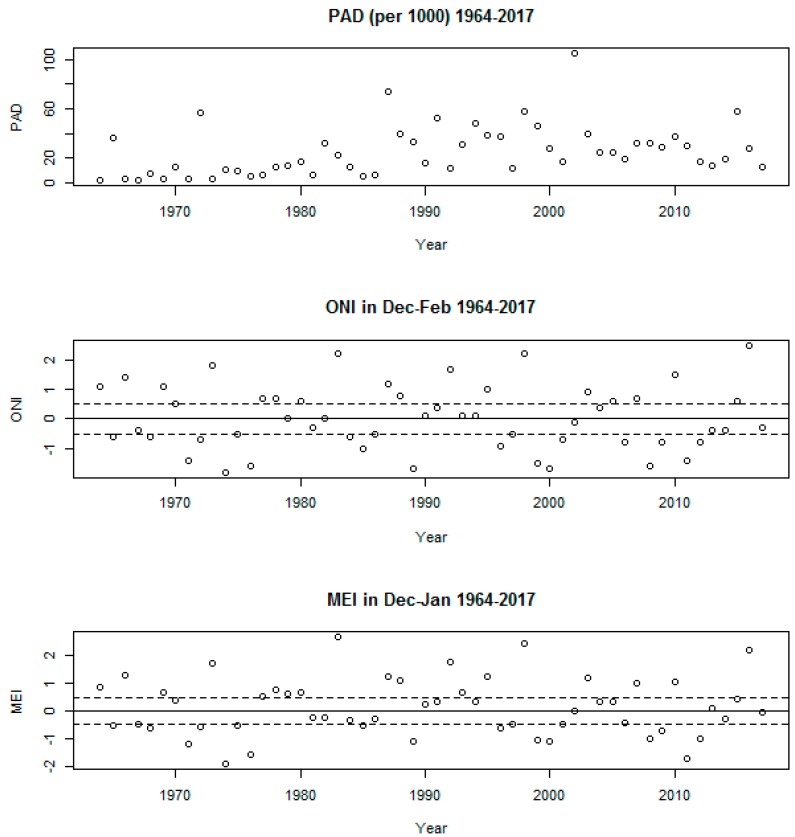
Annual time-series of ONI (DJF), MEI (DJ) and rates of people affected by natural disasters (PAD) (per 1000). Global. Yearly data from NOAA and EM-DAT, 1964–2017.

There was a general trend of increases in yearly overall PAD rates during the study period, although there has been a slight reduction in rates since the turn of the 21st century (Figure 1). A similar trend is observed with PAD rates specifically for floods, whereas the increasing rates associated with droughts and storms have slowed down in the last two decades (not shown). The graph shows that there have been no obvious increases in extremes of ONI and MEI over time. Strong signals of El Niño (ONI/MEI above 2) are apparent for 1983, 1998 and 2016 with the strongest La Niña signal (ONI/MEI close to −2) recorded in 1974.

**Figure 2:**
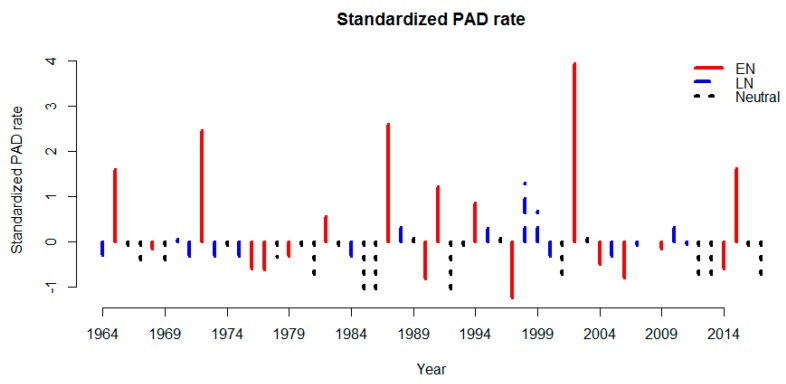
Standardized detrended global PAD rate by year, 1964–2017. Years indicated by solid line, dashed line and dotted line represent El Niño (EN), La Niña (LN) and neutral years [6], respectively.

**Figure A1:**
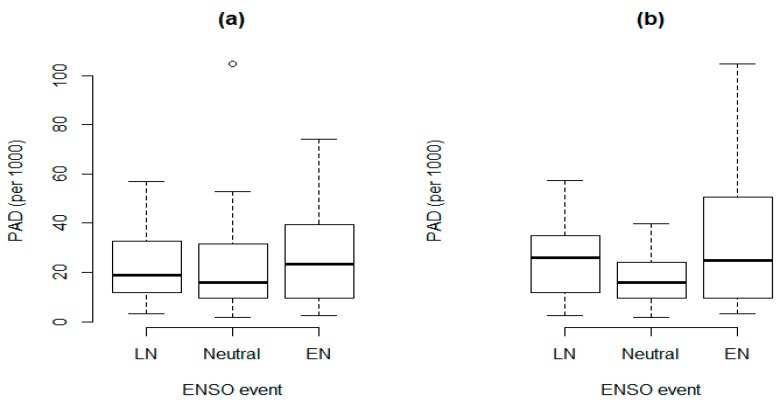
Global PAD rates by El Niño (EN), La Niña (LN) and neutral years, 1964–2017. EN, LN or neutral years defined as (**a**) type 1 events beginning in the second half of the previous year and ending in the first half of the same year as the disaster year; and (**b**) type 2 events beginning in the second half of the same year as the disaster year and ending in the first half of the following year. Events: ONI > 0.5 °C (for EN) or <−0.5 °C (for LN), otherwise neutral in five consecutive three-months overlapping seasons.

Figure 2 shows the standardized detrended PAD rate plotted against year. Out of the 54 years, seven years showed a standardized PAD rate >1: 1965, 1972, 1987, 1991, 1998, 2002 and 2015. The seven years have been marked as either El Niño or La Niña [6]. The plot reveals considerable inter-annual variability in PAD with several of the years displaying a standardised PAD rate of >1 being associated with strong (1965, 1972, 1987, 1991 and 2015) or weak (2002) El Niño events [6]. Figure A1 shows box-and-whisker plots of PAD rates subdivided by years that were either El Niño, La Niña or neutral years. El Niño (La Niña) years were defined as either: (a) type 1 ENSO events beginning in the second half of the previous year and ending in the first half of the same year as the disaster year; or (b) type 2 ENSO events beginning in the second half of the same year as the disaster year and ending in the first half of the following year. For type 1 events, median disaster rates were slightly higher in El Niño years compared to either La Niña or neutral years, while for type 2 events, both El Niño and La Niña years had higher PAD rates than neutral years, although the differences were not statistically significant in Kruskal-Wallis Tests.

The effect of ENSO on global PAD rates was most obvious in the same year. Table 2 shows same year-relative risks of global PAD rates at various intensities (defined as boundary points) of ONI and MEI compared to values of zero, after adjustment for long-term trends in PAD rates. The table suggests increasing PAD with increasing strength of ENSO indices with both warm (positive ONI/MEI) and cold (negative) phases, although confidence intervals were relatively wide and consequently trend tests non-significant.

**Figure 3:**
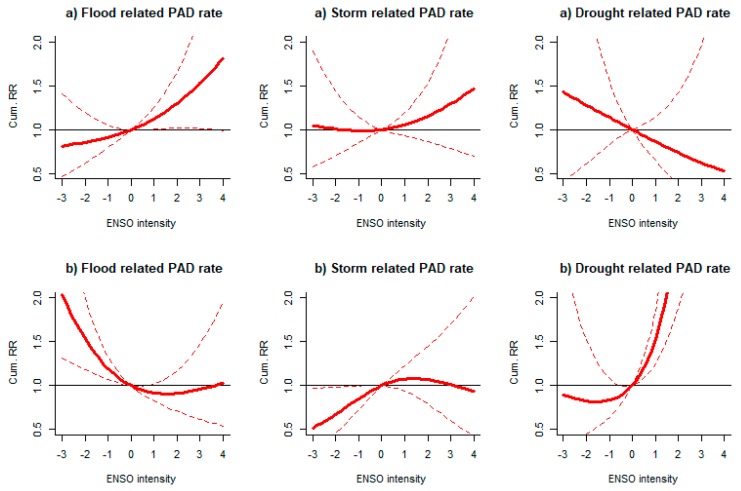
Relationship between global rates of PAD by specific disasters and ENSO intensity in the same year. Cum. RR: Cumulative relative risk. El Niño years defined as (**a**) type 1 events; and (**b**) type 2 events. Events: ONI > 0.5 °C (for EN) or <−0.5 °C (for LN), otherwise neutral in five consecutive three-months overlapping seasons. ENSO intensity: =4 for very strong (ONI ≥ 2 °C), =3 for strong (1.5–1.9 °C), =2 for moderate (1.0–1.4 °C) and =1 for weak (0.5–0.9 °C), and similarly for La Niña events based on negative values.

Same-year associations between disaster-specific PADs (floods, storms and droughts) and intensity of ENSO events at the global level were evaluated (Figure 3). When considering type 1 ENSO events, flood-related PAD rates increased during El Niño years (positive ENSO intensity) (Figure 3a). Flood-related PAD rates increased by 30% (95% CI 2%, 66%) during moderate El Niño years (indicated by ENSO intensity = 2 in Figure 3a; equivalent to ONIs between 1.0 °C and 1.4 °C in five or more consecutive overlapping three-month seasons) compared to neutral years (ENSO intensity = 0; equivalent to ONIs between −0.5 °C and 0.5 °C). When considering type 2 ENSO events, an opposite ENSO-flood-related PAD association was observed. Flood-related PAD rates increased during La Niña years (negative ENSO intensity) (Figure 3b). The flood-related PAD rate increased by 53% (95%CI 18%, 99%) during moderate La Niña years (indicated by ENSO intensity = −2 in Figure 3b; equivalent to ONIs between −1.0 and −1.4 in five or more consecutive overlapping three-month seasons) compared to neutral years. When considering type 2 ENSO events, a strong association was found between drought-related PAD rates and El Niño years. The PAD rates increased by 183% (95% CI 88%, 326%) during moderate El Niño years compared to neutral years (Figure 3b). No other comparisons were statistically significant, including those for storms or droughts.

### 3.3. Regional Analysis

In the regional analysis, PAD caused by floods and storms were evaluated in East Asia, South Asia and Southeast Asia.

**Figure A2:**
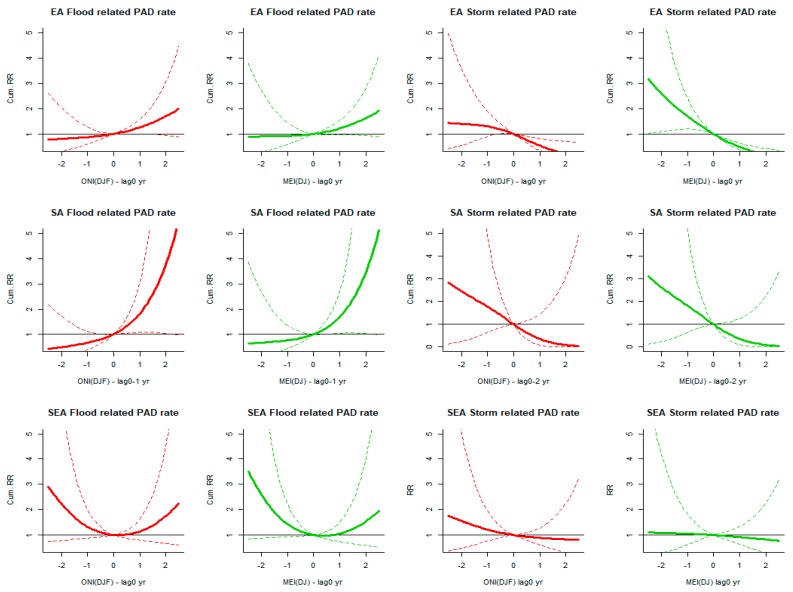
Relationships between ONI or MEI and flood- and storm-related PADs, by region of Asia (EA: Eastern Asia, SA: Southern Asia, SEA: Southeast Asia), 1964–2017. Solid lines represent the estimated relationship and dashed lines are 95% confidence limits.

For East Asia and South Asia, we found an El Niño/warm phase (ONI and MEI > 0.5) was associated with a higher flood-related PAD rates whilst higher storm-related PAD rates accompanied a La Niña or cool phase (ONI and MEI < −0.5) (Figure A2). In tests-for-trend across the range of ONI from −2 to 2, the risk of flood-related PAD rates increased by 14.0% (95% CI 3.9%, 25.1%) *p* = 0.014 for each boundary point increase (each 0.5 increase from −2) in Eastern Asia, and a 40.5% increase (19.3%, 65.6%) *p* = 0.002 in South Asia. For storms, boundary point increases in ONI (each 0.5 increase from −2) were associated with 23.3% reductions in PAD rate (14.7%, 30.9%) *p* = 0.001 in South Asia.

In sensitivity analyses, all global and regional analyses were repeated using the average of ONI within a year (from June-July-August in the previous year to May-June-July in the same year as the disaster year) to consider the importance of intensity of ONI in the 12 months rather than that in a single season, however all results were consistent with what was observed with the single season ONI (DJF) measure.

### 3.4. Country Analysis

**Figure A3:**
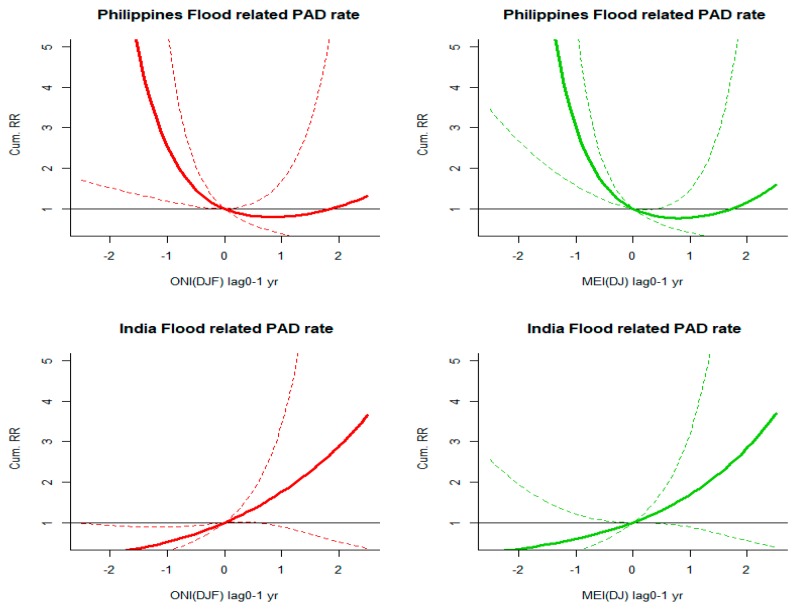
RR-DJF/RR-DJ plots for the association between PAD rate for flood and ONI(DJF)/MEI (DJ) in Philippines and India. Yearly data from NOAA and EMDAT, 1964–2017.

Among the nine flood-prone countries identified, PAD rates in the Philippines and India were found to be most sensitive to extreme ONI and MEI. The PAD rate in the Philippines increased during low ONI and MEI based on lags of 0–1 years (Figure A3). India had a higher risk of flood-related PAD rate with high MEI values at lags of 0–1 years. In a test-for-trend across the range of ONI from −2 to 2, flood-related PAD rates in India in the same year and the following year increased by 51.9% (25.5%, 83.9%) *p* = 0.002 for each boundary point increase (i.e., each 0.5 increase from −2). Thailand, China, Bangladesh and Sir Lanka had a high mean flood-related PAD rate, but no obvious associations were observed with ONI or MEI.

Among the eight storm-prone countries, the special administrative region of Hong Kong showed a higher risk of storm related PAD rate with low MEI values at lags 0–3 years. The Philippines was highly prone to storms with large numbers of people affected almost every year. Their risk of storm-related PAD rate increased when the ONI and MEI decreased for lags 0–2 years. Across the range of MEI from −2 to 2, storm-related PAD rates decreased by 33.1% (18.8%, 45.0%) *p* = 0.002 for each boundary point increase (each 0.5 increase from −2). Despite the high storm-related PAD rate in Vietnam, no obvious association was observed with either ONI or MEI in this study.

### 3.5. The Possible Modulation of ENSO-PAD Associations by the Pacific Decadal Oscillation

Understanding that the general nature of the two contrasting phases of ENSO are moderated by the Pacific Decadal Oscillation (PDO), a multi-decadal variation of warm and cool sea surface temperature conditions across the Pacific Basin [35,36,37,38], we investigated the same relationships as for the entire period (1964–2017), for the PDO warm phase 1975–2005. A complementary analysis for cool phase years (1964–1974 and 2006–2017) was not undertaken due to the small sample size. In general, the associations we found for the entire period strengthened during the warm phase, although these were less statistically significant than for the full time series due to the lower sample size (not shown). At the global level, analysis of MEI/ONI-PAD associations not only confirmed the importance of drought-related disasters associated with El Niño in the year of El Nino onset, but revealed that drought disasters are associated with El Niño in the following year, or the latter half of the ENSO cycle. At the regional level, the association with floods in East Asia was found to be different to that for the entire period of analysis such that flood-related PAD rates increased during La Niña instead of El Niño, which resonates with the general understanding of increased moisture levels over East Asia during La Niña years [39,40].

## 4. Discussion

Our results are suggestive of an association between El Niño and La Niña events and PAD at the global level, although with considerable heterogeneity according to disaster type. Flood events were the disaster type most strongly associated with El Niño, however flood burdens also increased in La Niña years when considering only impacts in the same year as the La Niña onset. Interestingly, the reverse was observed in the case of droughts, with increases associated with El Niño in onset years and also with La Niña in the year following onset. This may reflect the fact that the warm and cool phases have different lagged effects on flood- and drought- related PADs. In East Asia and South Asia, higher ONI and MEI values were associated with the risk of flood-related PAD rate, whilst lower ONI and MEI values were associated with higher storm-related PAD rates in all regions of Asia assessed. Although it might be assumed that periods of flooding are associated with storms, we did not observe significant correlations between storm- and flood-related PAD rates at the regional level nor consistent associations with ENSO events. This indicates that flood-related PADs in this study are unlikely to be storm-related. Storm types that do not bring large amounts of rain, such as sand or dust storms, might also have contributed to the different associations. However, the proportion of different storm types and their intensity could not be determined since a breakdown by disaster subtype was not available. The different associations with ENSO events may also indicate that other types of climate anomalies may contribute to PAD rates, such as the Indian Ocean Dipole (IOD) which has been found to moderate the relationship between ENSO and climate anomalies, especially over southern and southeast Asia [6,41].

The associations found were strongest in the same year as the El Niño event in East Asia, but in South Asia there was evidence that impacts were still present up to two years later. Of the countries assessed, India seemed most vulnerable to flood-related impacts during and following an El Niño event and the Philippines were most vulnerable to floods during and following La Niña. La Niña was also a problem in relation to storm-related PAD rates, in particular in Hong Kong. The links between La Niña and floods and storm impacts in this region is consistent with our understanding of La Niña events leading to anomalously wet conditions in regions of Southeast and East Asia, but the link between El Niño and floods is somewhat surprising as the likelihood of drier conditions in this region is expected to increase in El Niño years [6,15,42]. The influence of other physical climatic factors might have modified the expected ENSO effects [6]. For instance, the aforementioned IOD may amplify effects when it is in phase with ENSO but may also cancel out the effect when out of phase. This may be one of the contributing factors of the associations observed between flood-related PAD rate and El Niño in Southern Asia and India. The spatial-temporal variations among ENSO, other teleconnections and hazards may have also contributed to the inconsistency. For example it has been shown that ENSO-rainfall and IOD-rainfall associations vary spatially across mainland China and the South Asia region, respectively [15]. Also. there is evidence of non-stationary ENSO climate associations as revealed by the reversing direction of the correlation of ENSO and rainfall across southern China (negative tendency) and the Philippines (positive tendency) in recent years related to phase changes of the PDO [15]. Our subgroup analysis also indicated the reversed ENSO-flood association during the PDO warm phase in East Asia. In addition to the non-stationarity of ENSO-climate links, and by extension ENSO-PAD associations, the unique characteristics of each ENSO event [6] may well dictate the nature of the relationship between ENSO events and PAD outcomes—especially in relation to the longer lag effects considered in this study.

As with the Bouma et al. paper, this study found that populations in South Asia were generally at highest risk of ENSO-related disasters compared to other regions [19]. This was especially the case for impacts associated with floods following El Niño events and storms in the aftermath of La Niña. We also observed an El Niño association with drought-related PADs, however this was restricted to impacts in the same year as El Niño onset and little impact in subsequent years. This is in contrast to what has been found in one of the few other assessments of this nature where Dilley et al. reported that drought-related disasters are twice as frequent in the second year of an El Niño event compared to other years [13]. There may be a few reasons for the inconsistent results. First, the number of drought-related events in the EM-DAT database is relatively low for an annual level time-series analysis; this may have limited power to detect associations. Second, the different time periods considered in the studies may have given rise to some inconsistencies. We found that in the period 1964–1995, the number of drought events in all La Niña years (type 1 ENSO event) were below the entire study period median yearly number (median yearly number of droughts event = 11.5 in 1964–2017), whilst for the period 1996–2017, 7 La Niña years (type 1 ENSO event) were associated with drought events higher than the study period median. This suggests that La Niña is more likely to be associated with drought events over the recent 20-year period. This may explain the stronger El Niño-drought association reported in Dilley’s study which considered data before 1992 [13]. Furthermore, analysing different databases with non-identical event records may also explain the differing results.

This study made use of the EM-DAT dataset, which is the most comprehensive open-source dataset available recording disaster information globally over an extended timespan and with a low inclusion threshold (10 or more people died or 100 or more people affected). However, limited by the nature of the disaster database, only associations present at an annual level could be assessed which may be at a time scale beyond that required to detect PAD responses to within-year dynamics of ENSO. There are likely to be inconsistencies in how disasters are defined and recorded in the EM-DAT database, especially in relation to different types of storms and possibly flooding, which can be either fluvial or coastal/marine in origin. Perhaps most importantly, although our disaster data spanned over 50 years, statistical power was still a key limitation of this study. Associations were in many cases imprecise, which limited interpretation. In particular, missing information on drought events meant that they could not be assessed at the regional or country level. Similarly, power limited analysis to only three disaster types and only regions and countries in Asia, and no analysis at sub-national level for large countries like India. Countries which are known to be heavily impacted by ENSO, such as Peru and Ecuador in South America [43,44,45], were therefore not assessed.

There is also a danger of over-interpreting individual p-values when multiple significance tests are conducted; there was however consistency in associations within the different disaster types and between the different ENSO indices tested which increases our confidence of a possible signal. In disaster databases there are likely to be variations in the accuracy of reporting between countries and over time. This may explain some of the spatial and temporal trends observed in PAD rates, however such inconsistencies are unlikely to be associated with the ENSO cycle and so would not bias our findings.

This study used ONI to represent ENSO activity. This reflects only anomalies of sea surface temperature in the Niño 3.4 region. To inform suitability of our exposure variable and the robustness of our findings depending on the index considered, we also assessed all associations using the multivariable index MEI. The results for the two indices were highly consistent. Furthermore, we also considered the average of monthly ONI measures over a 12-month period in an attempt to capture the intensity of ENSO activity over a longer period, and again results were consistent. Other large-scale climate mechanisms that might modify the associations addressed here, such as the IOD which has been suggested to increase ENSO effects when in phase, and the Arctic Oscillation which has been shown to be associated with ENSO [46], were not considered in this yearly analysis. Such modes of climate variability and others therefore constitute good candidates for future analyses of the links between large scale climate mechanisms and PAD.

The relationships between the ENSO cycles and disaster-related health burdens demonstrated in this paper can help improve disaster preparedness strategies. Early warning systems already exist for climate-sensitive infectious diseases based on seasonal climate forecast models for some locations [47]. ENSO events can now be predicted with relative accuracy several months in advance, which allied with the associations revealed here, can inform early warning systems and other public health protection measures. Countries in South Asia, in particular India, could especially benefit from the knowledge that flood-related impacts will likely rise in the year following El Niño onset and so can instigate preparatory and alleviating measures [48]. This study also raises questions as to the nature of the ENSO-PAD association under climate change. Although it has been postulated that ENSO cycles may have become more intense due to climate change [49], we observed no evidence for increases in either ONI or MEI in recent decades from Figure 1. This agrees with van Aalst who concludes that “based on current climate models, global warming will most likely have relatively little influence on the frequency or intensity of El Niño and La Niña episodes.” [12]. However, recent projections of ENSO activity under greenhouse warming point, with a ‘medium’ level confidence, to more frequent El Niño events and exaggeration of current climate teleconnection patterns [21,49]. This holds implications for more severe ENSO-related disasters in the absence of adaptation and improvements in disaster risk reduction strategies.

## 5. Conclusions

This study suggests that ENSO plays a role, via climate related hazards, in determining health burdens—assuming that these are related to the number of people affected by disasters. This seems reasonable in view of the probable increased burden of mental ill-health following floods [50]. Given the valuable lead time that forecasts can now provide of impending El Niño events, such as the one that was detected at the end of 2018 and persisted throughout early 2019 in the Northern Hemisphere, our findings may flag those regions and countries most vulnerable, as manifested via high PAD, to periodic variations in large scale climate patterns associated with climate phenomena such as ENSO. Knowing about the nature of ENSO-PAD associations and how these may be moderated by low frequency climate variations such as the Pacific Decadal Oscillation will aid improvement of early warning systems and other protection measures designed to anticipate and minimise the health impacts associated with climate and weather-related disasters. However, such warning systems will need to be tailored to specific regions and countries, and possibly season, given the geographical and seasonal dependency of ENSO-climate and thus ENSO-PAD associations.

## Figures and Tables

**Table 1:** Descriptive statistics for the total number of people affected by ‘natural’ disasters (PAD) in all countries, Eastern Asia, Southern Asia and Southeast Asia. Yearly data from EM-DAT, 1964–2017.

Region	All Natural Disasters	Droughts	Floods	Storms
World				
Number of years with recorded event (N)	54	54	54	54
Median of yearly PAD rate (per 1000)	19.20	2.97	8.38	2.59
Median of yearly total PAD	123,262,882	16,932,171	43,923,263	14,924,902
Total PAD in all years	7,848,332,058	2,656,928,512	3,730,385,245	1,111,097,557
% of all global PAD burden		33.85%	47.53%	14.16%
Eastern Asia				
Number of years with recorded event (N)		23	49	52
Median of yearly PAD rate (per 1000)		10.74	5.20	1.42
Median of yearly total PAD		16,330,000	7,718,344	2,230,618
Total PAD in all years		534,250,000	2,065,818,428	502,885,480
Regional contribution to global PAD burden		20.11%	55.38%	45.26%
Southern Asia				
Number of years with recorded event (N)		24	53	54
Median of yearly PAD rate (per 1000)		1.65	11.73	1.25
Median of yearly total PAD		1,950,000	16,681,018	1,270,373
Total PAD in all years		1,478,885,000	1,306,601,275	189,308,058
Regional contribution to global PAD burden		55.66%	35.03%	17.04%
South-eastern Asia				
Number of years with recorded event (N)		26	52	53
Median of yearly PAD rate (per 1000)		3.79	3.39	6.34
Median of yearly total PAD		2,065,844	1,467,589	2,704,570
Total PAD in all years		77,006,716	161,721,850	228,053,018
Regional contribution to global PAD burden		2.90%	4.34%	20.53%

**Table 2:** Relative risks (RR) of global PAD rate associated with ONI and MEI in the same year as the disaster year, 1964–2017.

Intensities of ONI and MEI	ONI (DJF)	MEI (DJ)
ONI & MEI intensity in the same year as the disaster year (*N* = 50)	RR in same year (95% CI) (compared to ONI = 0)	RR in same year (95% CI) (compared to MEI = 0)
−2 (lower boundary for very strong)	1.14 (0.52, 2.51)	1.30 (0.54, 3.12)
−1.5 (lower boundary for strong)	1.09 (0.63, 1.88)	1.19 (0.65, 2.19)
−1 (lower boundary for moderate)	1.04 (0.76, 1.43)	1.10 (0.78, 1.59)
−0.5 (lower boundary for weak)	1.01 (0.88, 1.15)	1.04 (0.89, 1.21)
0.5 (lower boundary for weak)	1.02 (0.93, 1.13)	0.99 (0.89, 1.10)
1 (lower boundary for moderate)	1.07 (0.86, 1.33)	1.00 (0.81, 1.25)
1.5 (lower boundary for strong)	1.13 (0.79, 1.63)	1.04 (0.73, 1.49)
2 (lower boundary for very strong)	1.22 (0.71, 2.11)	1.10 (0.64, 1.88)

## Appendix A

**Table A1:** Descriptive statistics of the total number of people affected by natural disaster (PAD) and PAD rate (per 1000) by type and country/city with disaster events reported in not less than 30. Yearly data from EMDAT data, 1964–2017.

Disaster Type and Country Affected		*N* (Years)	PAD			PAD Rate (per 1000)		
			Mean	Standard Deviation	Total	Mean	Standard Deviation	Total
**Southern Asia**								
**Flood**	**Bangladesh**	44	7,550,661.11	11,492,162.74	332,229,089.00	76.92	126.12	3384.41
	**India**	48	18,079,598.83	21,096,396.54	867,820,744.00	19.97	23.49	958.42
	**Pakistan**	37	2,145,508.70	4,087,179.70	79,383,822.00	17.50	30.85	647.32
	**Sri Lanka**	38	424,075.66	363,428.43	16,114,875.00	23.72	21.17	901.54
**Storm**	**Bangladesh**	48	1,766,055.88	3,622,470.20	84,770,682.00	18.90	47.32	907.30
	**India**	45	2,211,721.60	3,811,566.05	99,527,472.00	2.63	4.62	118.25
**Southeast Asia**								
**Flood**	**Indonesia**	44	241,857.48	219,953.42	10,641,729.00	1.29	1.19	56.79
	**Philippines**	42	793,572.24	1,197,212.63	33,330,034.00	10.40	15.92	436.74
	**Thailand**	32	1,879,431.63	2,488,868.57	60,141,812.00	30.61	38.08	979.59
	**Viet Nam**	32	1,028,385.94	1,479,383.08	32,908,350.00	13.97	21.09	447.08
**Storm**	**Philippines**	52	3,168,649.98	3,701,601.92	164,769,799.00	42.93	40.23	2232.25
	**Viet Nam**	40	1,333,759.03	2,715,834.20	53,350,361.00	20.00	47.33	800.02
**Eastern Asia**								
**Flood**	**China**	38	54,112,338.82	65,199,287.36	2,056,268,875.00	42.61	51.86	1619.18
**Storm**	**China**	36	13,709,379.22	20,594,509.62	493,537,652.00	10.66	15.90	383.71
	**Hong Kong**	30	1932.43	3401.67	57,973.00	0.37	0.59	11.23
	**Japan**	41	58,561.39	84,776.22	2,401,017.00	0.47	0.69	19.43
	**Korea**	31	22,130.71	33,676.08	686,052.00	0.57	0.95	17.75
